# Supplementary material for: High resolution mapping of a novel non-transgressive hybrid susceptibility locus in barley exploited by P. teres f. maculata
Source: BMC Plant Biol. 2024 Jun 29;24:622. doi: 10.1186/s12870-024-05303-1 (PMC11218204; doi:10.1186/s12870-024-05303-1)
Supplement: Supplementary file 1 — Supplementary Material 1 [file 12870_2024_5303_MOESM1_ESM.docx]

## Supplemental Information

**Supplemental Information 1.** Amplicons utilized in the 365 SNP marker panel derived from the barley 9K iSelect platform for initial mapping in the CI5791 × Tifang F_2_ population. Marker names and sequences were obtained from T3 Barley.

**Supplemental Information 2.** Mapping data for the CI5791 × Tifang F_2_ population using the 9K derived PCR-Genotyping-By-Sequencing protocol. Individuals are in columns and marker sites are in rows. Zeros and twos represent CI5791 and Tifang respectively, whereas ones represent heterozygous calls. Phenotype of inoculation with 13IM8.3 is appended to the last row.

**Supplemental Information 3.** Amplicons derived from the barley 50K iSelect platform used for the high-resolution mapping in the CI5791 × Tifang F_2_ population. Marker names and sequences were obtained from T3 Barley.

**Supplemental Information 4.** Mapping data for the CI5791 × Tifang F_2_ population using the 50K derived PCR-Genotyping-By-Sequencing protocol. Individuals are in columns and marker sites are in rows. Zeros and twos represent CI5791 and Tifang respectively, whereas ones represent heterozygous calls. Phenotype of inoculation with 13IM8.3 is appended to the last row.

**Supplemental Information 5.** Amplicons derived from the barley exome capture data used for the high-resolution mapping in the CI5791 × Tifang F_2_ population. Marker names and sequences were obtained from exome capture information.

**Supplemental Information 6.** Mapping data for the CI5791 × Tifang F_2_ population using the exome capture derived PCR-Genotyping-By-Sequencing protocol. Individuals are in columns and marker sites are in rows. Zeros and twos represent CI5791 and Tifang respectively, whereas ones represent heterozygous calls. Phenotype of inoculation with 13IM8.3 is appended to the last row.

**Supplemental Information 7.** Mapping data for the CI5791 × Golden Promise F_2_ population using the exome capture derived Genotyping by Multiplex Sequencing protocol. Individuals are in columns and marker sites are in rows. Zeros and twos represent CI5791 and Golden Promise respectively, whereas ones represent heterozygous calls.

**Supplemental Information 8.** Primers for PACE assays used for the high-resolution mapping in the CI5791 × Tifang F_2_ population. Allele specific forward primers are labelled F1 and F2, whereas the common reverse primer is labelled CR. Primers to multiplex together are labelled by the PACE assay number.

**Supplemental Information 9.** Mapping data for the CI5791 × Tifang F_2_ population using PCR Allele Competitive Extension® assays. Individuals are in columns and marker sites are in rows. Zeros and twos represent CI5791 and Tifang respectively, whereas ones represent heterozygous calls. Phenotype of inoculation with 13IM8.3 is appended to the last row.

**Supplemental Figure 1.** Whole genome quantitative trait loci mapping of CI5791 × Tifang F_2_ population from 9K derived markers.

**Supplemental Figure 2.** Frequency plot of phenotypic disease reaction scores for each mapping round including 9K, 50K, exome capture CI5791 × Tifang F_2_ populations and GMS CI5791 × Golden Promise F_2_ population.
