# Supplementary figures and images for: High resolution mapping of a novel non-transgressive hybrid susceptibility locus in barley exploited by P. teres f. maculata
Source: BMC Plant Biol. 2024 Jun 29;24:622. doi: 10.1186/s12870-024-05303-1 (PMC11218204; doi:10.1186/s12870-024-05303-1)

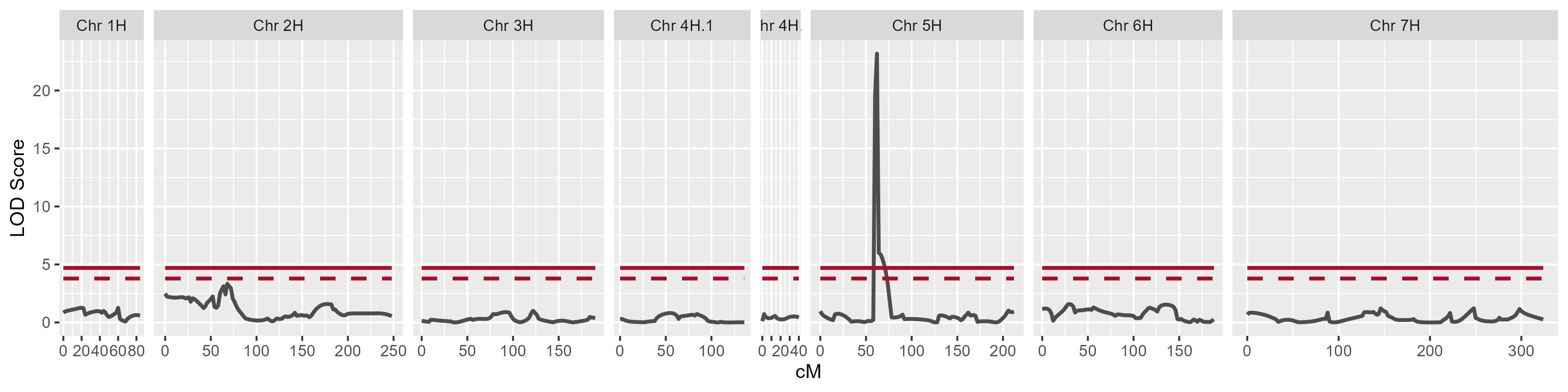

Supplement: Supplementary file 3 — Supplementary Material 3 [file 12870_2024_5303_MOESM3_ESM.jpeg]

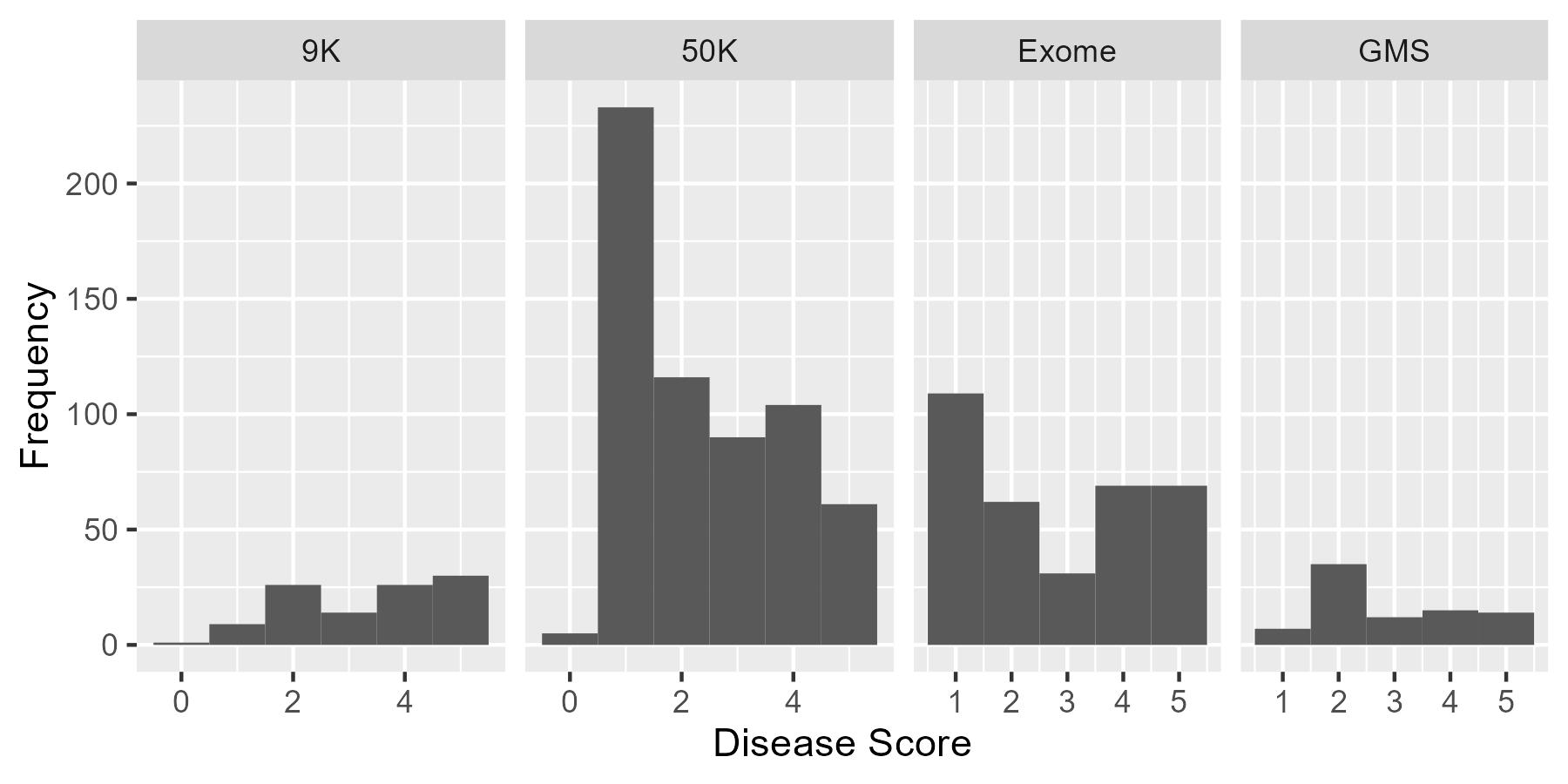

Supplement: Supplementary file 4 — Supplementary Material 4 [file 12870_2024_5303_MOESM4_ESM.jpeg]
